# Snare device retrieval of occluder embolized in patent foramen ovale closure: A case report

**DOI:** 10.1097/MD.0000000000038299

**Published:** 2024-05-31

**Authors:** Huijuan Hou, Qiang Zhang, Hongwei Dong

**Affiliations:** a Department of Cardiovascular Medicine, Dezhou Hospital, Qilu Hospital of Shandong University, Dezhou, China.

**Keywords:** occluder abscission, patent foramen ovale, transcatheter interventional closure therapy

## Abstract

**Rationale:**

Transcatheter interventional closure therapy is the main treatment method for patent foramen ovale (PFO). However, occluder abscission is a serious complication in PFO interventional therapy. Thus, timely and effective management of the occluder detachment is crucial for improving patient prognosis.

**Patient concerns:**

A 52-year-old female patient was admitted to the Department of Neurology mainly due to “right upper limb weakness for two days, which aggravated for eight hours.” The patient had no history of any other diseases.

**Diagnoses:**

The patient was diagnosed with an atrial septal defect (foramen ovale type) and cerebral infarction.

**Interventions and outcomes:**

The occluder abscission was successfully removed after several attempts with the help of the snare device.

**Lessons:**

When the PFO occlusion device is detached, interventional treatment would lead to minimal trauma, fast postoperative recovery, and a definite therapeutic effect. Based on mastering the indications and standardizing the operational process, this is a safe and effective minimally invasive treatment method.

## 1. Introduction

Patent foramen ovale (PFO) is a channel that connects the left and right atria due to failure of the primary and secondary atrial septum to close tightly after birth in infants, and this occurs in approximately 25% of the population.^[1]^ Some patients with PFO have been associated with diseases, such as stroke and migraine, and approximately 50% of cryptogenic stroke patients have concurrent PFO.^[2,3]^ At present, transcatheter interventional closure therapy is the main treatment method for PFO.^[4–6]^ Occluder abscission is a serious complication in PFO interventional therapy, which seriously threatens the life of patients. With the increasing number of patients undergoing PFO occlusion treatment, the risk of occluder abscission has increased from 0.4% to 3.0%.^[7]^ Therefore, timely and effective treatment options for occluder abscission can greatly improve the prognosis of patients. We report a case, in which the occluder abscission was successfully removed after several attempts with the help of the snare device.

## 2. Case presentation

A 52-year-old female patient was admitted to the Department of Neurology mainly due to “right upper limb weakness for two days, which aggravated for eight hours.” The patient had no history of any other diseases. No obvious positive physical signs were found in the heart, lungs, or abdomen. Blood pressure was 136/82 mm Hg. Muscle strength of the right upper limb was grade 4, while muscle strength of the rest of the limbs was grade 5. Muscular tension was normal in all limbs, and the tendon reflex of the limbs was positive (++). No pathological signs were found, and there were no significant abnormalities in the sensory or cerebellar examinations. The National Institute of Health Stroke Scale score was 1 point. The brain magnetic resonance imaging revealed multiple ischemic and infarct lesions in the brain, including a new infarction near the left lateral ventricles. The foaming experiment result was grade III (latent type). The trans-esophageal echocardiography revealed an increase in amplitude of activity of the atrial septal foramen ovale, with a tunnel-like separation and a length of approximately 8.2 mm. The continuous Doppler flow imaging revealed a left-to-right shunt signal at the atrial level, with a beam width of approximately 1.3 mm. The head and neck computed tomography angiography, and other auxiliary examinations did not reveal any abnormalities. The patient was diagnosed with an atrial septal defect (foramen ovale type) and cerebral infarction.

**Figure 1.**
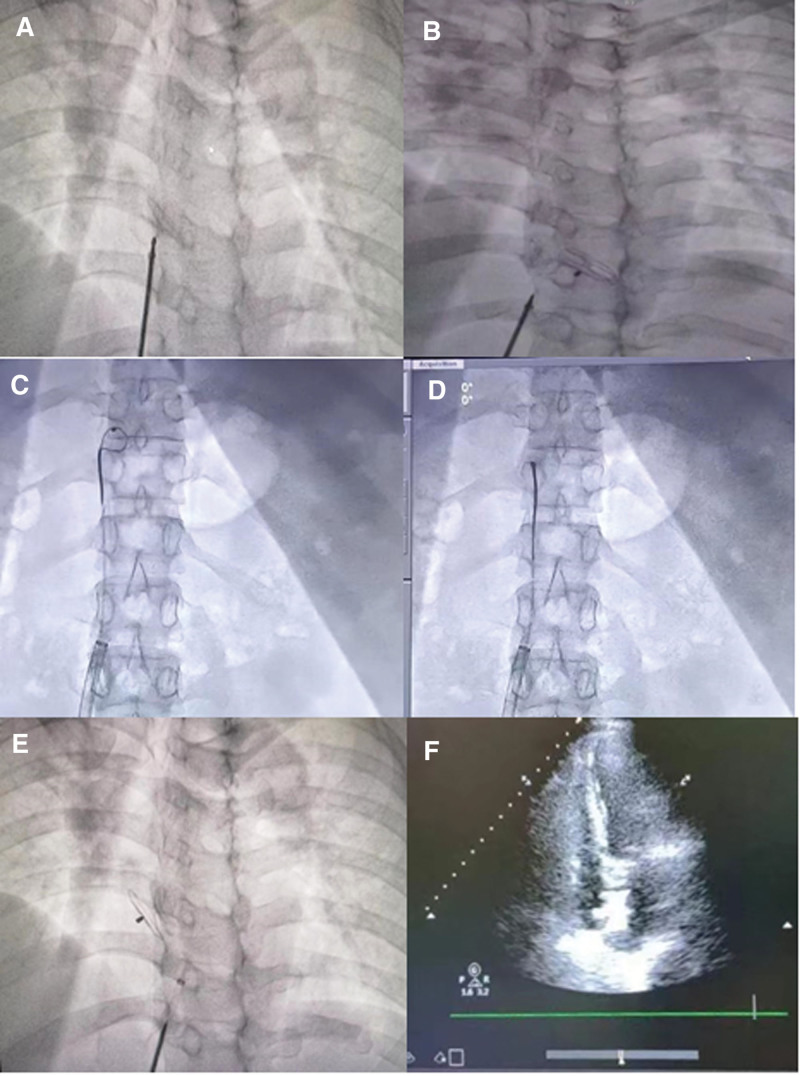
Closure, detachment, and interventional removal of the occlusion device during the patent foramen ovale. (A) The image before the occlusion device release. (B) The occlusion device detached after release. (C and D) The snare captured the single rivet end of the occlusion. (E) Successful closure of patent foramen ovale after interventional treatment. (F) The cardiac ultrasound revealed that the occlusion device was well-positioned.

After a comprehensive evaluation, the patient agreed to undergo interventional closure treatment in the cardiology department. The oxide film single-riveted atrial septal defect occluder (DMFQFDQ-I 24, Shanghai Shape Memory Alloy Co., Ltd., Shanghai) was selected. After the occluder was released in the correct position, it was observed to be detached under fluoroscopy (Fig. 1A). Then, this fixed to the pulmonary artery after entering the right ventricle through the right atrium (Fig. 1B). Burst ventricular tachycardia was observed by electrocardiographic monitoring, followed by recovery of sinus rhythm. The patient’s vital signs were stable, and a 5F Cordis VER135° angiographic catheter (100 cm, 0.038”) was immediately employed in conjunction with a J-shaped super-slick guidewire to reach the pulmonary artery. The snare-20 (Shanghai Xingji, Shanghai) was applied.

After repeated attempts, the snare was successfully placed on the waist of the occlusion device, and the occlusion device was retracted to the inferior vena cava together with the contrast catheter and delivery sheath. After several attempts, it was not possible to drag the occlusion device into the delivery sheath. Therefore, the occlusion was released into the inferior vena cava, and a 16F sheath was used as a replacement. The waist of the occlusion was caught again. However, without the elastic deformation, the occlusion could not be retracted into the larger sheath. Hence, the Weixin WXSK-20 snare was employed to capture the single rivet end of the occlusion (Fig. 1C and D). The 16F long sheath was held in place with both hands, and the end of the snare was grasped using a needle holder. With force, the occlusion was drawn into the larger sheath, and successfully withdrawn from the body.

The new oxide film single-riveted atrial septal defect occluder (DMFQFDQ-I 24, Shanghai Shape Memory Alloy Co., Ltd.) was replaced, the foramen ovale interventional occlusion treatment was performed again, and the occlusion was successful (Fig. 1E). The follow-up echocardiography revealed good positioning of the occlusion device at 1 day postsurgery (Fig. 1F). The femoral vein puncture site was sutured after the operation. At 4 days postsurgery, the patient reported no chest pain or tightness. The physical examination revealed stable vital signs, with a blood pressure of 120/80 mm Hg and a heart rate of 70 beats per minute, displaying a regular rhythm. The respiratory sounds in both lungs were clear, with no detected abnormal lung sounds, such as dry or wet rales. The cardiac sound was normal, with no murmurs detected upon auscultation of the heart valves, or at the puncture site of the right femoral vein. In addition, the arterial pulsations in the feet were strong, and there was no evidence of swelling in the lower extremities. As a result, the patient was discharged from the hospital. The follow-up cardiac ultrasound performed at 6 months after surgery confirmed the proper positioning of the occlusion device.

## 3. Discussion and conclusions

Occluder abscission usually occurs during occlusion surgery, with a few cases occurring within hours to days after surgery.^[8]^ This is often caused by selecting an occluder that is too small, the special anatomical location of the lesion, an improper operation, or the quality of the equipment itself. The occluder can detach in the left atrium or right atrium, but this more commonly occurs in the right atrium. Occluder abscission often leads to arrhythmia, and in severe cases, this can lead to ventricular fibrillation.^[9]^ If conditions permit, the occluder can be attempted to be removed via catheterization. In general, this is pulled out of the body through a large-diameter delivery sheath via the venous route. If unsuccessful, emergency surgery should be performed to remove the occluder, and close the atrial septal defect.^[10]^ This intervention method would cause minimal trauma to patients, but this remains challenging, and has a low success rate.^[11]^ The present case report describes how the investigators successfully used interventional methods to remove a detached occluder, providing valuable experience for clinical treatment.

In the present case, the occluder immediately detached after release during the surgery, and was immobilized in the pulmonary artery from the right atrium to the right ventricle with blood flow. Since the occluder was small, and had a mesh structure, this did not affect the hemodynamics. This provided the surgeon with sufficient time to use interventional methods to retrieve the occluder. In terms of occluder selection, the investigators chose the oxide film single-riveted atrial septal defect occluder. The occluder has a rivet structure on the release surface for easy capture. The occluder was first withdrawn from the pulmonary artery to the inferior vena cava, and the waist of the occluder was captured using a snare, in order to avoid triggering malignant arrhythmias through excessive manipulation in the right ventricle. The snare captured the rivet end of the occluder, allowing it to recover its prerelease compressed state. If the snare captures the waist of the occluder, the occluder would not be able to recover its elastic deformation, and cannot be recovered into the sheath due to its large umbrella surface. If the rivet structure cannot be captured, other techniques, such as the percutaneous transluminal coronary angioplasty (PTCA) guidewire used in previous studies, can be applied to retrieve the occluder.^[12]^ However, the application of the PTCA guidewire would inevitably damage the structure of the occluder. There have been reports of snare devices being used to capture the waist of the occluder abscission, and recover it into the sheath.^[13]^ However, this is only applicable to smaller umbrella occluders for ventricular septal defects. For the present case, the sheath was changed to 16F before retrieving the occluder using the snare device. This reduced the resistance, and prevented further detachment. After the snare device captured the rivet end of the occluder, the snare device was fixed with needle holders, and withdrawn at a uniform speed. The occluder retrieval process required the external segment of the delivery sheath to be firmly secured, and the withdrawal force must be moderate, in order to prevent damage to the snare device, and further detachment of the occluder.

In the present case, it was found that due to the different cardiac positions of the patient, the occluder could not be released according to the anatomical position considered by conventional experience. Before release, ultrasound and imaging data should be used to determine the position of the occlusion device, and repeated traction tests should be performed to further confirm its position. When encountering detachment of the occlusion device, the retrieval procedure should be selected based on the patient’s hemodynamic performance. For patients who are hemodynamically stable without severe arrhythmias, interventional methods can be initially attempted to remove the occlusion device. When the condition becomes unstable, an emergency thoracotomy is necessary. The instruments used in interventional treatment, such as snare devices, PTCA guidewire,^[12]^ angling guidewire,^[14]^ and endoscopic forceps,^[15]^ have been reported in the literature. However, the specific choice of instruments should be based on the location and shape of the detached occlusion device. For the present case, a snare device was used to capture the head of the occlusion device for retrieval. Although the occlusion device can be restored to its prerelease state, the high resistance of small-diameter sheaths can increase the risk of device detachment. Therefore, the investigators still recommend the replacement of the sheath with a larger diameter, in order to shorten the operation time, and increase the success rate of the operation. The specific size of the sheath that can successfully retrieve the occlusion device, while minimizing damage to the peripheral blood vessels, still requires further clinical experience.

It is imperative to recognize the constraints inherent in the present case. Although interventional treatment presents potential in occluder abscission, it is crucial to conduct further assessments, incorporate extended follow-up periods, and have a more extensive patient cohort, in order to thoroughly evaluate its efficacy and complication rates across time.

When the PFO occlusion device is detached, the interventional treatment would lead to minimal trauma, fast postoperative recovery, and a definite therapeutic effect. Based on mastering the indications and standardizing the operational process, this is a safe and effective minimally invasive treatment method.

## Author contributions

**Data curation:** Huijuan Hou.

**Formal analysis:** Huijuan Hou.

**Writing—original draft:** Huijuan Hou.

**Resources:** Qiang Zhang.

**Visualization:** Qiang Zhang.

**Investigation:** Hongwei Dong.

**Methodology:** Hongwei Dong.

**Project administration:** Hongwei Dong.

**Writing—review & editing:** Hongwei Dong.
